# Graphene-Like-Graphite as Fast-Chargeable and High-Capacity Anode Materials for Lithium Ion Batteries

**DOI:** 10.1038/s41598-017-14504-8

**Published:** 2017-11-01

**Authors:** Qian Cheng, Yasuharu Okamoto, Noriyuki Tamura, Masayoshi Tsuji, Shunya Maruyama, Yoshiaki Matsuo

**Affiliations:** 1 IoT Devices Laboratories, NEC Corporation, Tsukuba, Ibaraki, 305-8501 Japan; 2 Department of Applied Chemistry, Graduate School of Engineering, University of Hyogo, Himeji, Hyogo, 671-2280 Japan

## Abstract

Here we propose the use of a carbon material called graphene-like-graphite (GLG) as anode material of lithium ion batteries that delivers a high capacity of 608 mAh/g and provides superior rate capability. The morphology and crystal structure of GLG are quite similar to those of graphite, which is currently used as the anode material of lithium ion batteries. Therefore, it is expected to be used in the same manner of conventional graphite materials to fabricate the cells. Based on the data obtained from various spectroscopic techniques, we propose a structural GLG model in which nanopores and pairs of C-O-C units are introduced within the carbon layers stacked with three-dimensional regularity. Three types of highly ionic lithium ions are found in fully charged GLG and stored between its layers. The oxygen atoms introduced within the carbon layers seem to play an important role in accommodating a large amount of lithium ions in GLG. Moreover, the large increase in the interlayer spacing observed for fully charged GLG is ascribed to the migration of oxygen atoms within the carbon layer introduced in the state of C-O-C to the interlayer space maintaining one of the C-O bonds.

## Introduction

Among energy-storage technologies, lithium ion (Li-ion) batteries have become critical for a variety of applications from portable electronics to electric vehicles because of their relative high energy density, power density, and long cycle life. Recently, electric vehicles, drones, and transport robots with autonomous capabilities, such as autopilot, have attracted much interest as state-of-the-art technologies. For widespread use of these technologies, however, batteries with only high energy density are not sufficient as power sources for these applications since fast charging capability, long cycle life, and low cost are also essential. A further increase in both energy density and power performance has become very limited in the last two decades since there has been little progress in commercializing new chemistry for significantly higher capacity, fast-charging, and discharging capability^1–5^.

Silicon and transition metal oxides have been considered as anode materials of Li-ion batteries due to their high theoretical capacity^6–8^. However, the extremely high volume change (~400%) experienced during lithiation/delithiation results in pulverization of the active material and loss of electrical connection^6,9,10^. In addition, the solid electrolyte interphase (SEI) layer, which is formed during the 1st cycle and has to bear the same volume expansion and contraction, will also crack and delaminate from the Si, leading to a very thick SEI layer after several cycles, leading to rapid capacity loss. Although some researchers attempted to use a core-shell structure^11–13^, porous^14–17^, or hollow silicon sphere^18^ to solve these problems, it is still far from real application. Regarding fast-charging capability, a porous Si anode was reported with 2-C charging capability, but the density is quite low, which is not practical^19^. Based on the above reasons, carbon materials are currently the most popular commercialized anode material for Li-ion batteries because of its relative high capacity, long cycle life, low cost, and ease of processing. However, the limited theoretical capacity (372 mAh/g) and small interlayer space (0.335 nm) makes it difficult to use in the application of Li-ion batteries with higher energy density and fast chargeability.

In this context, graphene, as the parent of all graphitic structures, has been studied as an anode material exhibiting high capacity and good rate performance^20–25^. However, graphene is rarely used for battery electrodes because of its low density and high specific surface area, which result in low initial coulombic efficiency. The material density, which is much lower than 0.8 g/cc, is not favorable for use in a battery with high volumetric energy density^25,26^. However, we previously reported that thermal reduction of graphite oxide (GO) provide carbon materials with large interlayer spacings but low surface area, maintaining the high regularity of the orientation of graphene layers^27–30^. This was achieved only when the temperature-increasing rate during thermal reduction of GO was low enough to avoid its exfoliation. When thermally reduced GO were used as anodes of Li-ion batteries, the shape of the charge-discharge curves were similar to those recently reported for graphene-based materials. Interestingly, the thermal reduction of GO showed a interlayer space of 0.34 nm, which is a little bit larger than that of pristine graphite; however, the interlayer space of thermally reduced GO increased 0.12 nm after full lithiation^30^. This value was much larger than that (0.03 nm) observed for graphite with the same charge state. As a result, some of the thermally reduced GO samples showed a much improved capacity of 580 mAh/g^28,30^. Therefore, we concluded that Li ions are stored on both sides of graphene layers in them. More recently, similar materials have been used for the electrode of electric double-layer capacitors exhibiting higher capacity and rate capability^31,32^. Larger ions, such as tetrafluoroborate (BF_4_^−^) and tetramethylammonium ((CH_3_)_4_N^+^), are also intercalated, which is considered resulting from the reduced van der Waals energy between adjacent graphene layers. These indicate that the carbon layers in this material act as if they are independent graphene, although they are regularly oriented as those in graphite^33,34^. Therefore, we call this material “graphene-like-graphite” (GLG). We also reported on the intercalation of Na ions into GLG with an interlayer spacing of 0.34 nm, which was very similar to that of graphite, and the residual oxygen in the form of C-O-C facilitated the reduction of GLG^35,36^. In this study, we investigated the structure, electrochemical properties of GLG in detail and proposed a structural model of GLG in which nanopores and pairs of C-O-C units are introduced within the carbon layers stacked with 3 dimensional regularity.

## Experimental section

### Synthesis of GLG

We synthesized GO from natural graphite (Z-5F, Ito Graphite) by oxidation with KClO_3_ in fuming HNO_3_ for 3 h at 60 °C based on Brodie’s method^37,38^. The obtained GO was then heat treated in a step heating process. Specifically, GO was heated under vacuum from room temperature to 170 °C at a temperature-increasing rate of 1 °C/min then heated to 250 °C at 0.1 °C/min. After that, the temperature-increasing rate was changed to 1 °C/min until it reached 800 °C. The temperature was kept at 800 °C for 5 h, then a small amount of air (ca. 15 kPa) was introduced in the reactor and naturally cooled. The obtained sample was used without any further treatment.

### Characterization

We examined the morphology of the products by using field emission scanning electron microscopy (FE-SEM) (Hitachi, SU8000, 5 kV) and conducted X-ray diffraction experiments at room temperature under specular reflection mode. Measurement was carried out with a Rigaku Geigerflex diffractometer (Cu Kα radiation; 40 kV, 20 mA). The STEM sample was prepared by dropping the ammonia GO dispersion (0.05 mg/mL) onto an amorphous C-coated TEM grid. The resulting GO samples were heated in a TEM chamber at 800 °C to convert them to GLG. A HAADF-STEM image was created using JEOL JEM-ARM200F Dual-X with accelerating voltage of 80 kV and 0.2 nmφas the beam diameter. The image was also processed by deconvolution treatment. Temperature programmed desorption-mass spectrometry (TPD-MS, GC/MS QP2010plus10) was used to analyze the gaseous products emitted from GLG when heating it from room temperature to 1000 °C at 10 °C/min in He atmosphere. Rutherford backscattering spectrometry (RBS, National Electrostatics Corp. Pelletron 3SDH) was used for the depth analysis of O and C by 4He^++^ with input energy of 2300 keV.

Hard X-ray photoelectron spectroscopy (HAXPES) measurement was carried out at BL46XU of SPring-8. The electronic analyzer was R4000 manufactured by VG SIENTA. Measurement was conducted at room temperature, with 200 eV as the pass energy and a curved 0.5-mm slit. The take-off angle of the photoelectrons was set at 80°.

The potential range for the half-cell measurement was from 0 to 2 V. The GLG samples in the 1st and 2nd cycles were investigated by high-resolution solid-state ^7^Li-NMR. The samples were washed with pure diethyl carbonate (DEC) solvent to remove electrolyte salt then vacuum dried. We obtained ^7^Li-NMR spectra using a JEOL ECA600 (233.2491 MHz for ^7^Li nucleus) spectrometer with magic angle spinning and single pulse mode. Spinning speeds of 19 and 21 kHz were achieved for all samples. We repeated scanning 4096 times to improve the signal-to-noise ratio of the spectra. We also conducted *ex-situ* X-ray diffraction measurement of GLG electrodes charged or discharged at various levels using the Bruker D2-Phaser (CuKα; 20 kV, 10 mA). The samples were removed from the electrochemical cell in an Ar-atmosphere glove box and washed with dimethyl carbonate (DMC). They were then placed in a sealed sample holder to avoid contact with air.

### Cell fabrication and electrochemical tests

The electrochemical properties of the samples were characterized in both half-cell and full-cell configurations. The potential range for initial charge and discharge was from 0–2 V. For half-cell measurement, Li metal and 1 M LiPF_6_-ethylenecarbonate (EC) + DEC (3:7) or 1 M LiClO_4_−EC + DMC (1:1) without additives were used as counter electrodes and electrolyte solution, respectively. Lithium nickel manganese cobalt oxide (NMC111, BASF) was used as a cathode for full-cell measurement.

A negative electrode was prepared by coating a mixture of an active materials (either GLG or pristine graphite), carbon black, water-based binder carboxymethyl cellulose, and styrene-butadiene rubber with a weight ratio of 92:3:2:2 on a copper foil with a mass loading of 50 g/m^2^ for a single side. The density of both reference graphite and GLG electrodes was adjusted to 1.15 g/cc. The size of the anode was 23 × 24 mm. The cathode electrode was prepared by coating a mixture of NCM111, carbon black, and polyvinylidene fluoride with the weight ratio of 93:3:4 on an aluminum film with mass loading of 110 g/m^2^ for a single side. The density of the cathode electrode was 2.25 g/cc, and it was 22 × 23 mm in size. The full cells were assembled with as prepared anode and cathode with the anode/cathode ratio of 1:2. The cells were charged (Li-ion intercalation) to 0 V at 0.1, 0.2, 0.5, 1, 2, 3, 5, and 10 C then discharged (Li-ion deintercalation) to 1.5 V at 0.1 C. The full-cell charge and discharge were carried out at voltages of 2.5 to 4.2 V. In both cases, the GLG electrodes were charged using a constant-current and constant-voltage method then discharged using a constant current method.

### Computational Methods

All our first-principles calculations were done using the Quantum Espresso (ver. 5.0.2) program package. Calculations of the electronic structure were done in accordance with the DFT framework under the periodic boundary conditions with the vdW-DF2-C09 exchange correlation functional. All cell parameters (a, b, and c) and all ionic positions in the computational cell were fully relaxed in all calculations by using the Broyden-Fletcher-Goldfarb-Shanno algorithm. Ultrasoft pseudopotentials and Troullier-Martins-type soft pseudopotential were used for C (C.pbe-rrkjus.UPF) and Li (Li.pbe-mt_fhi.UPF), respectively. Note that the pseudopotential of Li includes the nonlinear core correction. Plane-wave basis sets with cut-off energies of 30 and 300 Ry were respectively used for the expansion of wave functions and charge density. We used 3 × 3 × 2 k-point samplings with Methfessel-Paxton smearing for Brillouin-zone integration.

## Results and Discussion

### Characterization of materials

**Figure 1:**
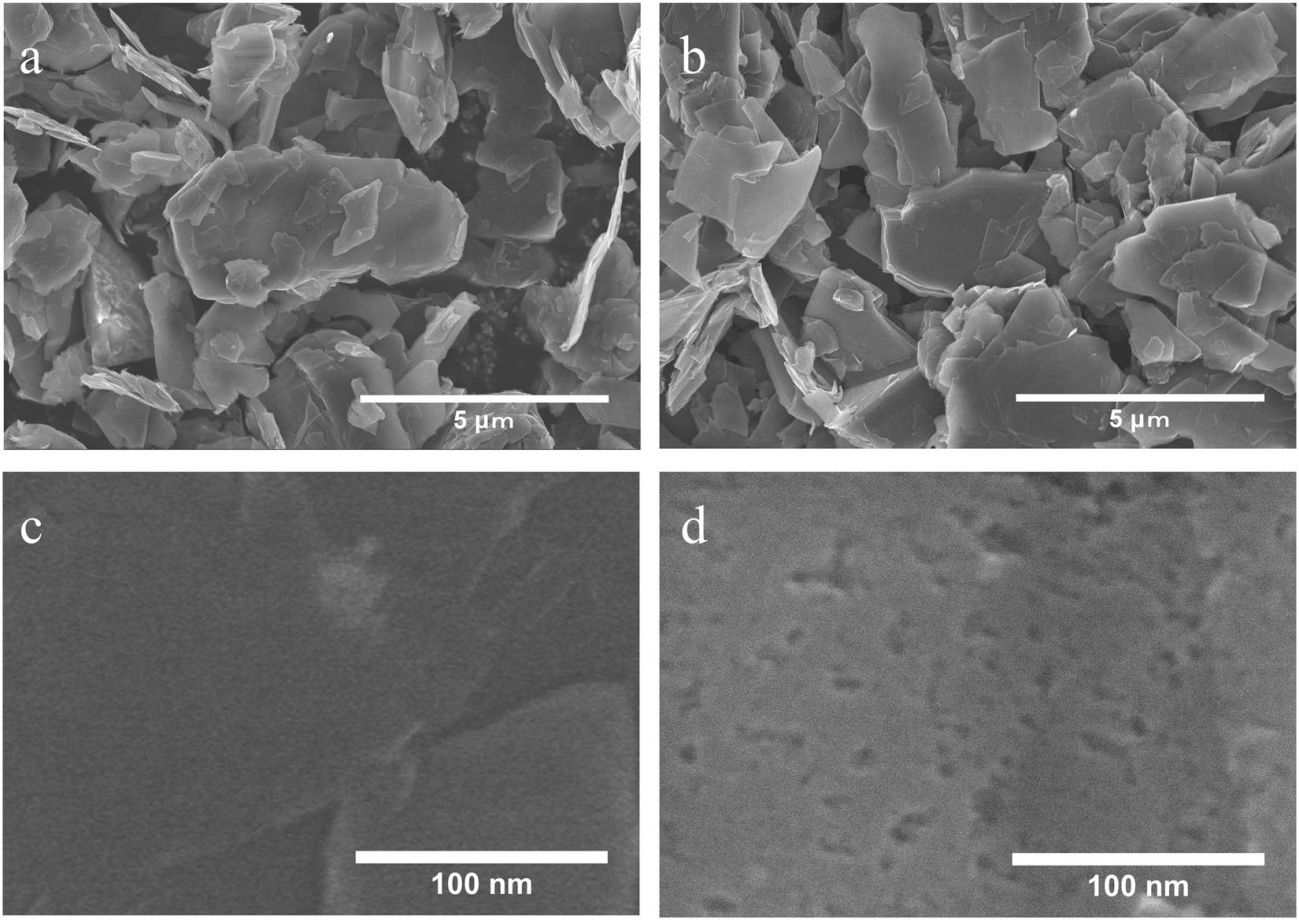
SEM images of pristine graphite and GLG. (**a**) SEM image of pristine graphite (**b**) SEM image of GLG. (**c**) High resolution image of pristine natural graphite surface. (**d**) High resolution image of GLG surface.

The morphologies of the carbon materials are shown in Fig. 1. Figure 1a is an SEM image of pristine graphite. Flake-type graphite around 5 μm was used for this experiment. Figure 1b is an SEM image of GLG at the same magnification to compare it with that shown in Fig. 1a. The GLG material has almost the same morphology and size as the pristine graphite in contrast with the graphene materials obtained from the rapid thermal reduction of GO^39^. As a result, the GLG is compatible with current graphite anode electrode fabrication process. Currently, researchers are attempting to control graphite, which is used as the anode material of Li-ion batteries, with a relatively small specific surface area to have an acceptable initial charge coulombic efficiency and cycleability. This is because there is no solvent that can survive without reduction at an ultra-low potential when charging to 0 V vs. Li/Li^+^, and an SEI will be formed on the surface of the anode materials, which consumes the Li from either the cathode or electrolyte^1,36,37^. Moreover, the required binder quantity is in proportion to the specific surface area of the electrode materials. Anode materials with high surface area, such as graphene (400–1200 m^2^/g), require 15 wt% of binder to fabricate an electrode, which makes it difficult to fabricate a battery with high energy density^21^. As a result, GLG with unaltered size is quite compatible with the current process to fabricate a Li-ion battery electrode. Figure 1c is an SEM image of pristine graphite surface, and Fig. 1d is the surface morphology at the same magnification of GLG. Micropores of 3–5 nm were revealed in GLG, while there were no pores on the surface of pristine graphite. The pores were supposed to be formed during the thermal-reduction process, in which O-containing functional groups of GO reacted with C atoms to form CO or CO_2_. The surface nanopores increase the specific surface area. Based on the N_2_-adsorption isotherms of pristine graphite and GLG shown in Fig. S1, the BET specific surface area of GLG was 31.3 m^2^/g, which was slightly larger than that of pristine graphite (11.9 m^2^/g). The GLG material is expected to have good initial coulombic efficiency due to its smaller specific surface area than graphene^31^.

**Figure 2:**
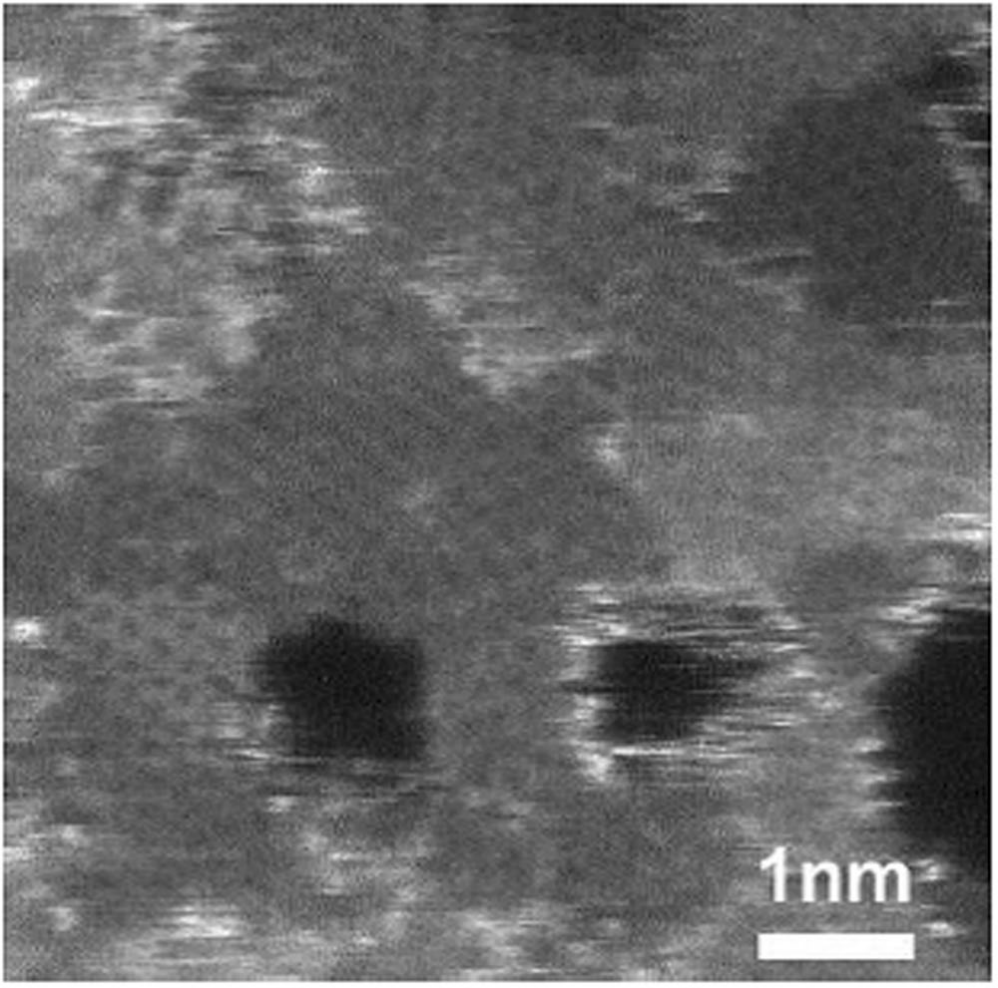
STEM image of GLG.

Figure 2 shows a high-resolution STEM image of exfoliated GLG. Nanopores of around 1 nm existed on the GLG graphene layers. This indicates that nanopores exist not only on the top-most surface of GLG, as shown above, but also inside each graphene layer.

**Figure 3:**
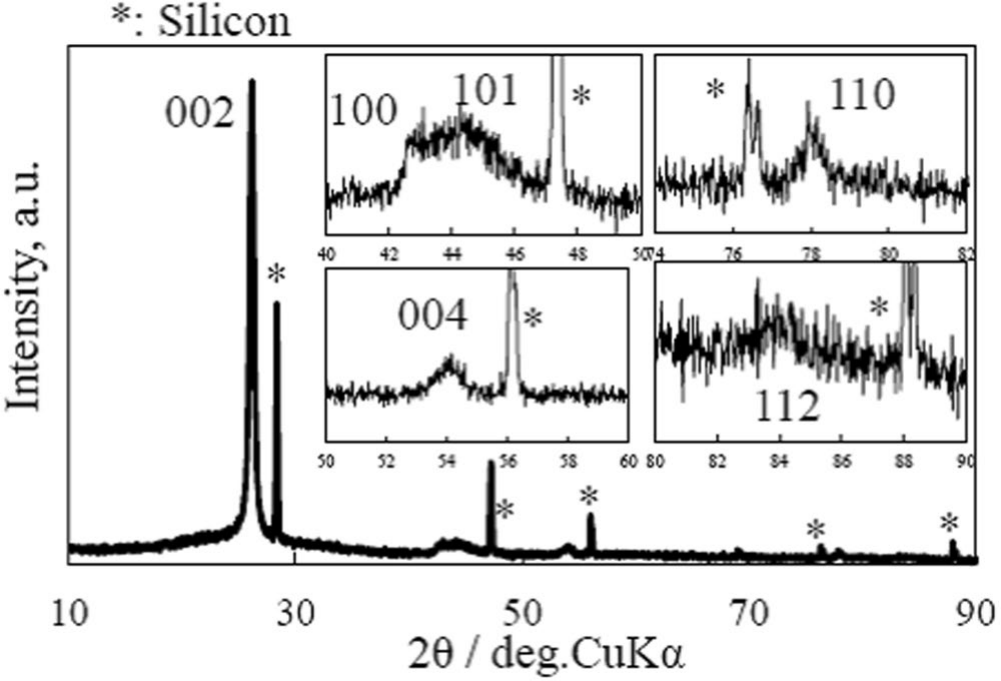
XRD pattern of GLG. Insets show magnified patterns in selected regions.

Figure 3 shows the XRD pattern of GLG. The (002) peak of GLG has a shift to the lower angle of 2θ = 26.26° (d = 0.339 nm) compared with that of graphite of 2θ = 26.57° (d = 0.3354 nm), which indicates a larger interlayer space of GLG. The (004) and (110) peaks were also observed at 2θ = 54.18 (d = 0.1693 nm) and 77.93° (d = 0.1226 nm).

The crystal sizes along the a- and c-axes (La and Lc) of GLG were estimated from the width of the (002) and (110) peaks, and 16.3 and 23.1 nm were obtained, respectively. They decreased dramatically from those of graphite (573 and 118 nm) after being converted to GLG. This is because the starting material for GLG is GO, which has many O-containing functional groups. During the thermal reduction of GO, the removal of C atoms also occurred, together with that of O-containing functional groups; therefore, discontinuous graphene defects in GLG should be introduced and the crystal sizes become much smaller.

However, surprisingly, together with the above peaks, separated (100) and (101), and (112) peaks at 2θ = 42.64 (d = 0.2120 nm), 44.38 (d = 0.241 nm) and 84.9° (d = 0.1142 nm) were observed. This means that three-dimensional stacking regularity of the carbon layers was still maintained in GLG, as in graphite. This result indicates that it is quite appropriate to call this material GLG.

**Figure 4:**
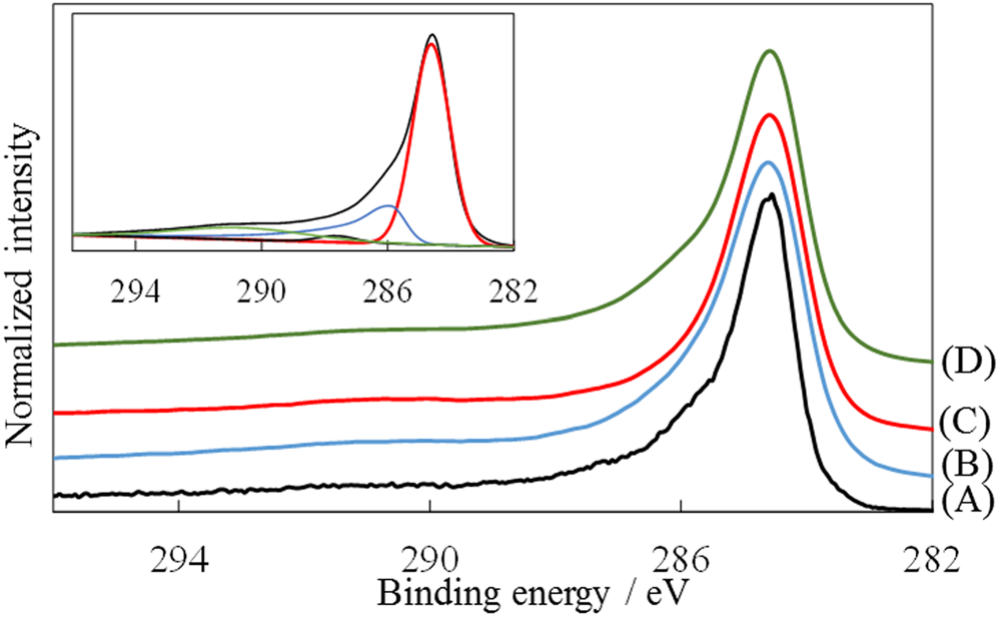
XPS spectra of (**A**) graphite, (**B**) GLG, and (**D**) exfoliated GLG, together with (**C**) HAXPES spectrum of GLG. Inset shows deconvoluted spectrum of exfoliated GLG.

The O content was measured as 6.3 wt% by elemental analysis. The XPS and HAXPES data of graphite, GLG, and exfoliated GLG in the C1s region are shown in Fig. 4. In the wide-scan spectra, only the peaks due to C and O were observed. The observed peaks were deconvoluted into four peaks at 284.7, 286.1, 287.9, and 291.1, which are assigned to C-C and/or C=C, C-O, C=O, and COO^−^, respectively^40^. Table 1 summarizes the composition of materials estimated from the area of the deconvoluted peaks. The main functional groups in GLG contain C-O bonds. The GLG material has an O content of 7.7 wt%, which was much higher than that of pristine graphite (0.6 wt%) when estimated from the XPS datum. However, the information depth in XPS analysis is on the order of a few nanometers, and the O content inside the GLG particle is unknown. Therefore, we also carried out HAXPES analysis of which the information depth reaches 50 nm. The obtained O content was 5.5 wt%, which was slightly less than that estimated from the XPS result. This indicates that the O content is higher at the surface area and slightly lower inside particles. The GLG material was also exfoliated to a few layers by sonicating it in NMP solution for 30 min at 10 W, and the resulting dispersion was dropped on a Si wafer. The composition of the resulting exfoliated GLG was rather similar to that of pristine GLG estimated from HAXPES analysis.

**Table 1:** Percentage of the peak area at 284.7, 286.1, 287.7 and 291.1 eV observed in XPS or HAXPES spectra of graphite, GLG and exfoliated GLG (10% as error for both XPS and HAXPES).

Sample	Method	Percentage of the peak area/%			
		284.7 eV	286.1 eV	287.9 eV	291.1 eV
Graphite	XPS	95	3	2	<1
GLG	XPS	89	8	3	1
GLG	HAXPES	91	7	2	<1
Exfoliated GLG	XPS	91	7	2	<1

**Figure 5:**
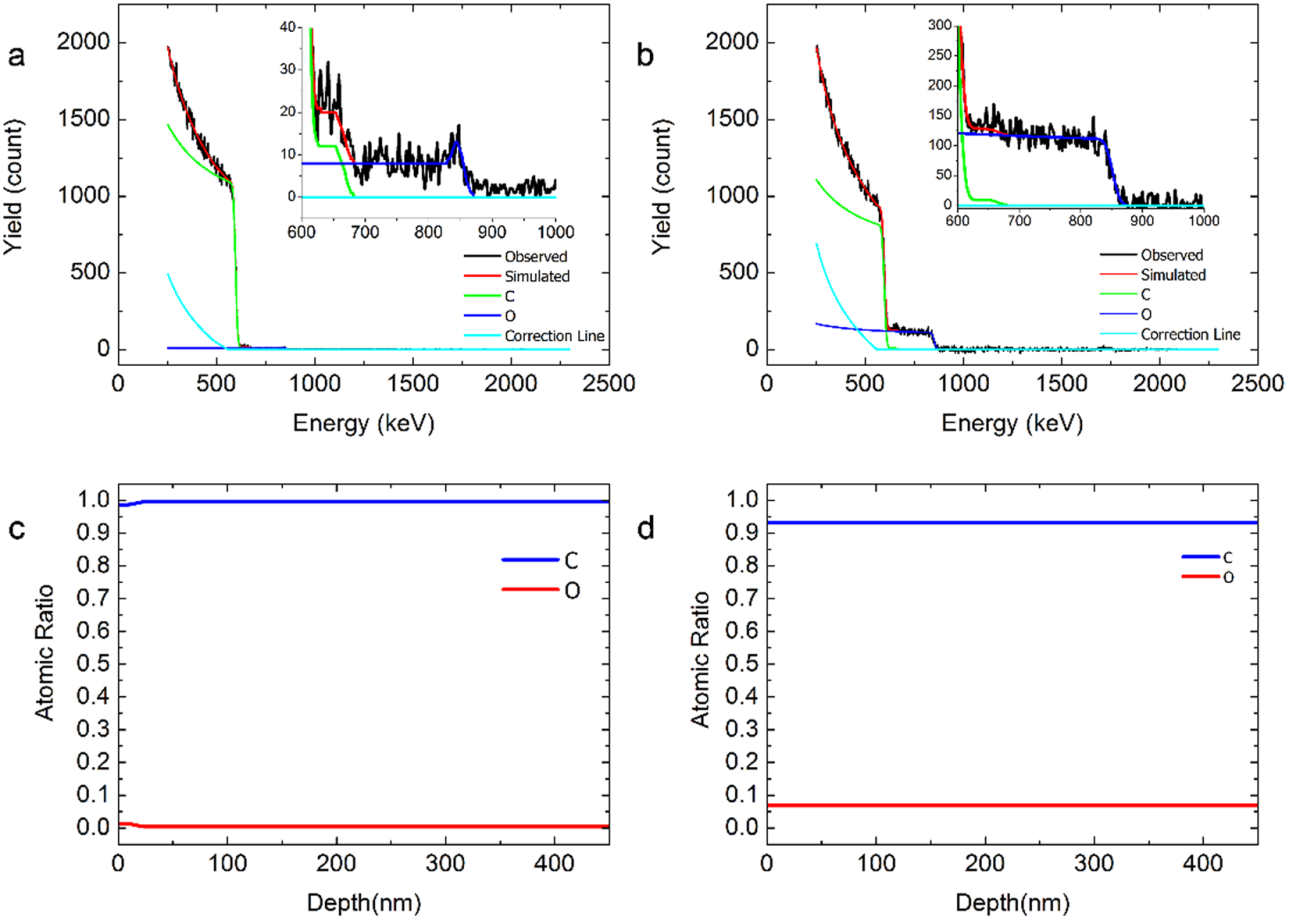
Rutherford back-scattering-spectrometry elemental-depth analysis of (**a,c**) pristine graphite and (**b,d**) GLG.

To determine the distribution of O within GLG particles more quantitatively, RBS measurement was also conducted, as shown in Fig. 5. For graphite, the yield of back-scattered He^+^ ions started to slightly increase around 845 keV (Fig. 5a). This means that pristine graphite has only a little O within less than 20 nm from the surface (Fig. 5c). For GLG, however, the yield greatly increased below 844 keV and became constant until the energy of back-scattered He^+^ ions reached 624 keV (Fig. 5b). The inset figures show the depth profiles of C and O estimated from the RBS spectra, indicating a uniform distribution of O throughout the entire GLG particle (Fig. 5d).

**Figure 6:**
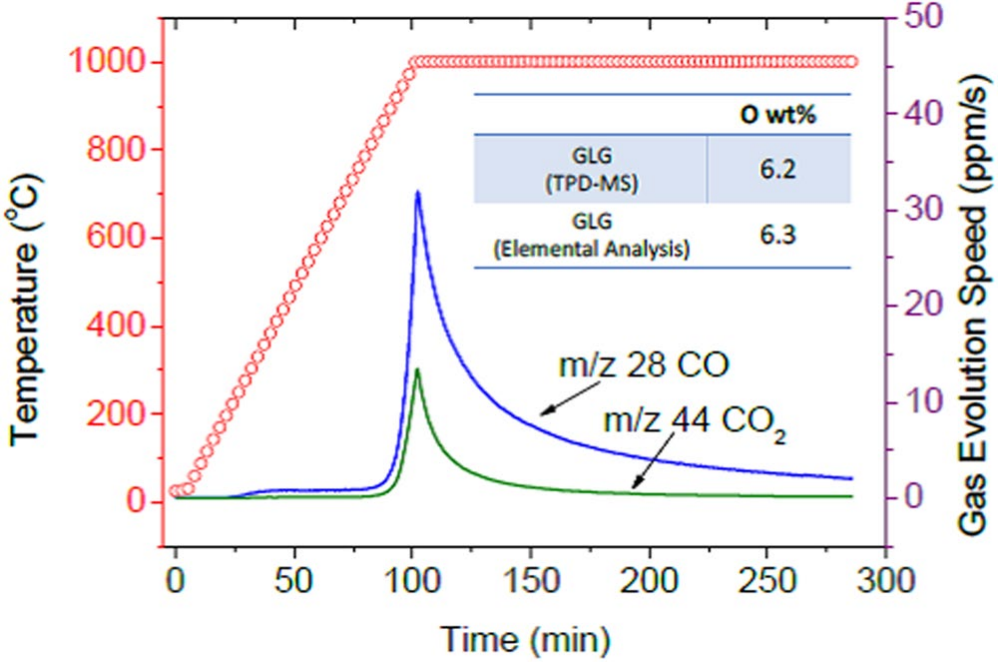
TPD-MS analysis of GLG. Inset table shows compositions calculated based on TPD-MS and elemental analysis data.

Temperature-programmed desorption coupled with mass spectroscopy (TPD-MS) was conducted to analyze the chemical composition of GLG (Fig. 6). The CO (m/z 28) and CO_2_ (m/z 44) were detected, and the total mass fractions of each gas were 8.5 and 1.8 wt%, respectively, after heating to 1000 °C in 100 min and maintaining at 1000 °C for 180 min in He atmosphere. The evolution of CO started around 300 °C and increased sharply from 800 °C. It has been reported that the CO gases evolve from carboxyl, anhydride and lactone groups at low temperatures of 100–400, 350–623, 290–650 °C, respectively^41,42^. The gas evolution at 100–400 °C could be ascribed to the decomposition of carboxyl groups, which was detected by XPS, as shown above^42,43^. The origin of the CO_2_ gases that evolved above 800 °C is rarely assigned to either carboxyl, anhydride, or lactone groups. Provided that nanopores exist within the carbon layers of GLG, as shown above, the functional groups that formed on their edges (e.g. lactone) may be more thermodynamically stable than those at the outer edge and decompose at higher temperatures above 800 °C. However, the evolution of CO gases start around 800 °C. They evolve from phenol, carbonyl, anhydride, ether, and quinone groups at 600–700, 600–980, 350–623, 700, and 700–980 °C, respectively^41,42^. Since the signal derived from C-O bonding was clearly observed in XPS measurement and H_2_O was not detected above 400 °C, ether groups may be the sources of CO gases. This supports the previously reported X-ray absorption results, which indicated that GLG contains ether groups introduced within carbon layers^44^. As described above, the thermal stability of these ether groups could also be higher and decomposed above 800 °C. The mass fraction of O calculated from the total amount of CO and CO_2_ was 6.2 wt%, which is well correlated with the value calculated from the elemental analysis datum (6.3 wt%).

**Figure 7:**
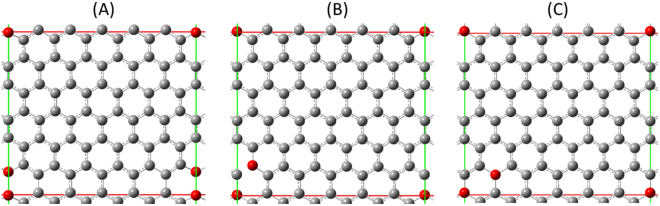
Local structure models of GLG with compositions of C_70_O_2_. (**A**) Ortho. (**B**) Meta. (**C**) Para.

Based on the data obtained from the above analyses and our previous results in which this material mainly contained C-O-C groups^36^, we considered how these groups are distributed within a carbon layer. The heat of formation was calculated based on density-functioned calculation of C_70_O_2_ with three arrangements, ortho (Fig. 7A), meta (Fig. 7B), and para (Fig. 7C), of O. The calculated values are −1.12, +4.32, and +4.68 eV, respectively. The reaction of C_70_O_2_ + C_72_ → 2C_71_O is endothermic by 5.43 eV, which means that C_71_O is thermodynamically quite unfavorable. This indicates that the C atoms should be pairwisely substituted with O atoms. As shown in Fig. 7, the distance between O atoms is 0.224 nm, which means that the O-O bond is actually broken in (A). As a result, O is expected to be C-O-C among graphene layers. This type of orientation of C-O-C groups introduced within graphene layers has been observed in graphene oxide by using TEM^44^.

**Figure 8:**
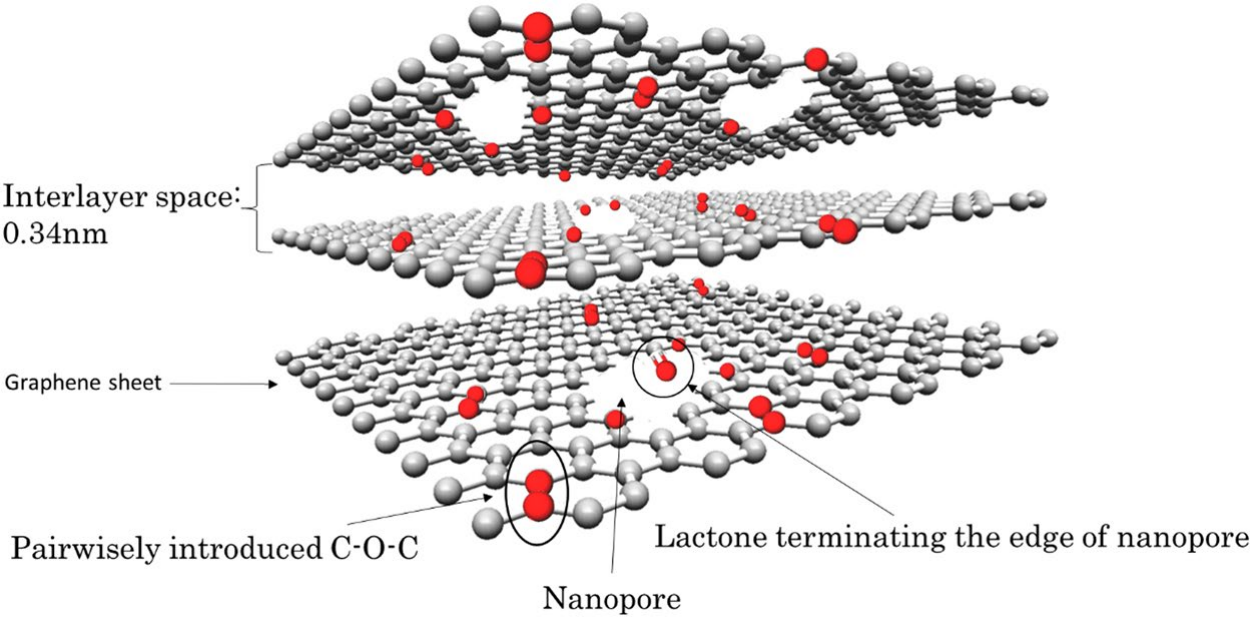
Schematic image of GLG.

We propose the structure model of GLG shown in Fig. 8. The GLG material contains 6.3 wt% of O atoms, even in its bulk. Most of the O atoms are pairwisely introduced within the graphene sheets in the form of C-O-C. Nanopores of 1–5 nm formed not only at the surface of GLG but also in its bulk. Some parts of the edges of the nanopores terminated with lactone groups considering the evolution of CO_2_ at higher temperatures as observed in TPD-MS measurement. The O-atom- and nanopore-introduced graphene sheets are regularly stacked with an interlayer space of 0.34 nm, which is quite similar to that of pristine graphite. Since either of the introduction of large amounts of oxygen or the small interlayer space of around 0.34 nm was observed for our previously reported carbons obtained from the thermally reduction of GO under hydrogen gas flow at higher temperatures^29,30^ or, under vacuum or hydrogen gas flow at 300 °C^27,28^, the present GLG material is novel.

### Electrochemical properties

**Figure 9:**
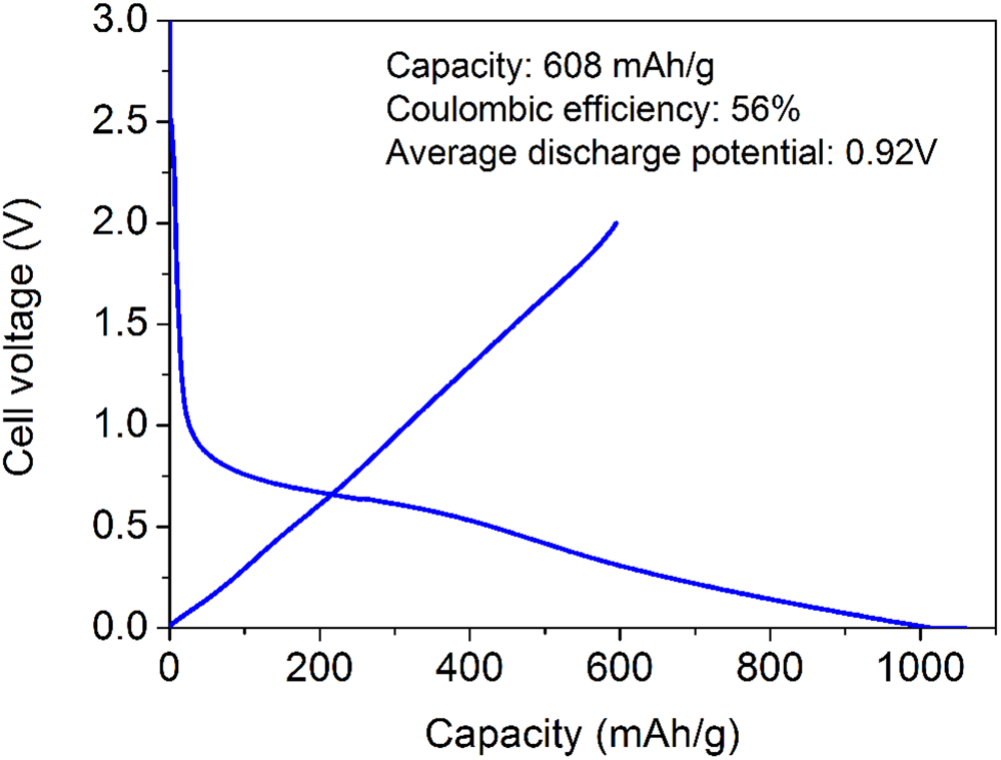
Charge and discharge curves of GLG.

Figure 9 plots the initial charge and discharge curves of GLG. During the charge, a plateau around 0.7 V was observed, then the electrode potential gradually decreased. During the discharge, the plateau increased almost linearly to 2 V. This behavior is similar to that reported for graphene-based materials^22,45^. The charge and discharge capacity was 1033 and 608 mAh/g, respectively. This discharge capacity of GLG is much larger than that of graphite and those of the GO thermally reduced under hydrogen gas flow at various temperatures^27,29,30^ or under vacuum at 300 °C^28^. The larger capacity is ascribed to the large amount of oxygen atoms introduced within carbon layers. The contribution of oxygen atoms in GLG materials on their capacities will be reported in detail in our next paper^46^. Many researchers have reported that graphene-based anodes exhibit larger discharge capacities, even exceeding 1000 mAh/g^21,45^; however, they exhibit higher discharge average potentials than that of GLG-based anodes, which was 0.92 V. The contribution of the capacity above 2 V, which is not available in real batteries, is quite large in graphene-based anodes. Graphene discharge capacities below 2 V are comparable or even smaller than that observed for the present GLG. The coulombic efficiency of GLG was 56%, which was much higher than those of graphene^21,45^. The lower coulombic efficiency may be attributed to 1) higher specific surface area (11.9 m^2^/g → 31.3 m^2^/g) and nanopore structure (Fig. 1d) compared with pristine graphite and 2) the strong interaction between Li ions and O-containing functional groups, which is attributed to the capacity in high potential (>1.5 V) discharge. Many Li ions may be still trapped by the functional groups even in 2-V discharge. The capacity and columbic efficiency increased to 670 mAh/g and 65%, respectively, when the cut off voltage was 3 V. The charge and discharge curve of conventional graphite is shown in Fig. S16 with the coulombic efficiency of 92%. The full cell charge and discharge curve of GLG as an anode and NCM111 as a cathode was shown in Fig. S17.

**Figure 10:**
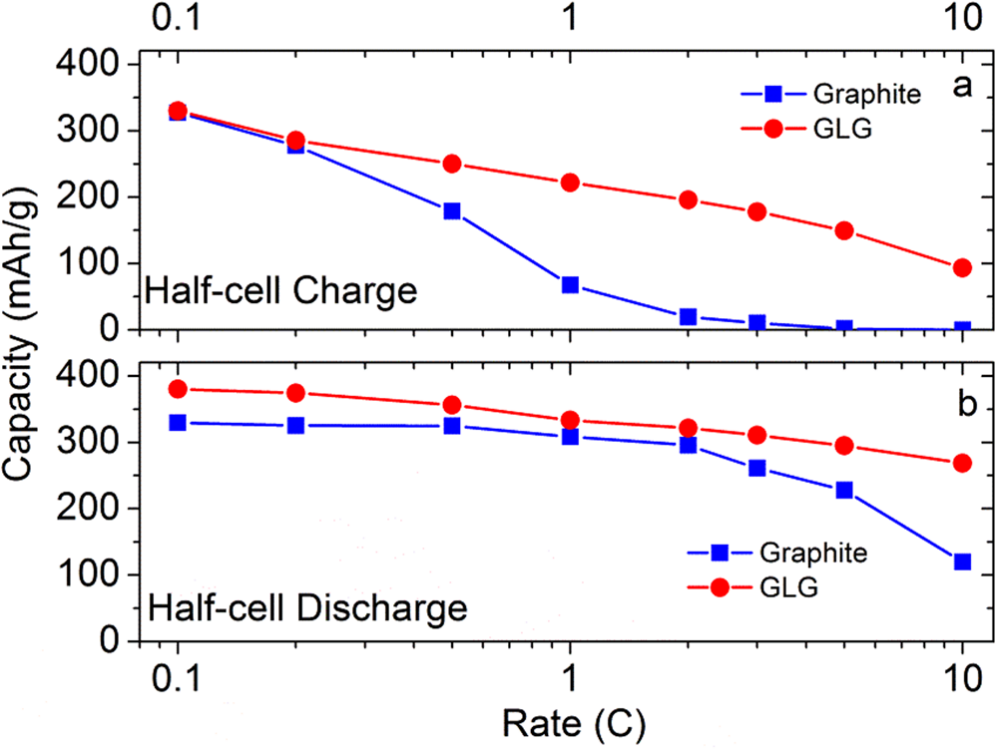
Half-cell (**a**) charge- and (**b**) discharge-rate capability of GLG in comparison with those of graphite.

The half-cell charge- and discharge-rate capabilities of pristine graphite and GLG measured are shown in Fig. 10. Figure 10a is the charge (lithiation)-rate capability of graphite and GLG. The cells were charged (Li-ion intercalation) to 0 V vs. Li metal at 0.1, 0.2, 0.5, 1, 2, 3, 5 and 10 C then discharged (Li-ion deintercalation) to 1.5 V vs. Li metal at 0.1 C from 1.5 to 0 V. The capacity at every C-rate is plotted. The GLG material exhibited much larger capacity at 0.5 C or higher, and even at 10 C, 30% of capacity was still delivered, which means that it has a better charge-rate capability. The half-cell charge and discharge curves of GLG at 0.1 C, 0.2 C, 0.5 C, 1 C, 2 C, 5 C, and 10 C were shown in Figs S14–S15. Figure S14 shows the Li-out rate capability of GLG. The IR drop increased with higher C-rate and the capacities higher than 0.8 V decreased faster in high C, which indicate the capacities from O containing carbon tend to worse rate capability. Figure 10b plots the discharge (delithiation)-rate capability of pristine graphite and GLG. The cells were charged (Li-ion intercalation) to 0 V vs. Li metal at 0.1 C and discharged (de-intercalation) to 1.5 V vs. Li metal at 0.1, 0.2, 0.5, 1, 2, 3, 5, and 10 C from 0 to 1.5 V. The excellent rate performance of GLG is attributed to the following. 1) Surface nanopores, which are characterized by SEM (Fig. 1d), provide extra intercalation or deintercalation sites for Li ions. 2) The internal pores, which are characterized by TEM (Fig. 3), of exfoliated GLG can facilitate the Li-ion diffusion rate. 3) The larger inter layer space (Fig. 4) facilitates a better diffusion rate during charging and discharging. 4) Smaller crystal size decreases the intercalation/deintercalation pathway for Li ions. As a result, GLG exhibited a superior rate performance than pristine graphite. The EIS curves of graphite and GLG are shown in Fig. S18.

**Figure 11:**
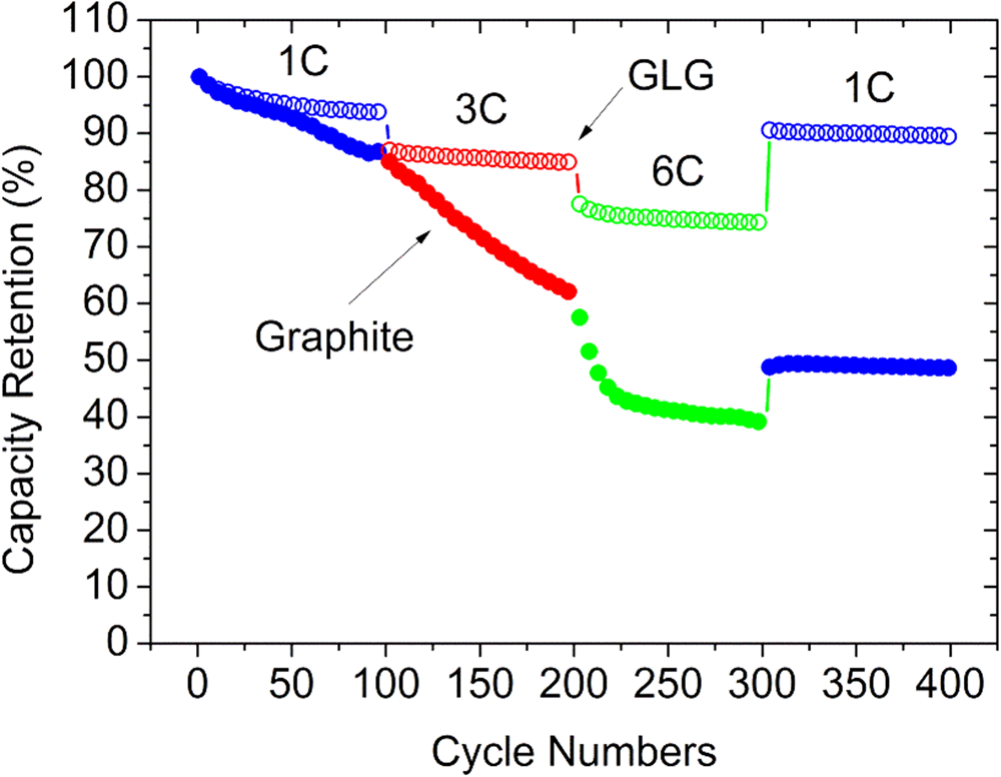
Cyclability of graphite and GLG in full cells operated at various rates.

Figure 11 shows the full-cell cyclability of graphite and GLG operated at 1, 3 and 6 C then 1 C. Graphite and GLG exhibited a capacity retention of 87 and 93%, respectively, after the first 100 cycles at 1 C. During the following 100 cycles at 3 C, the capacity of the cell with the GLG anode was almost constant and slightly dropped to 85% at the end of the 100 cycles, while that of graphite significantly decreased to 59%. The capacity retention of the cell with the GLG anode further dropped to 79% at 6 C; however, the capacity was again almost constant during the subsequent 100 cycles. However, the capacity of the cell with the graphite anode greatly decreased to reach 39% after another 100 cycles at 6 C. Finally, both cells were cycled at 1 C again for 100 cycles, and the cell with the GLG anode showed a capacity retention of 90%, in contrast, that of the cell with the graphite anode was only recovered to 49%. Thus, GLG exhibits a much better cycleability at high rate than pristine graphite.

### Mechanism analysis

**Figure 12:**
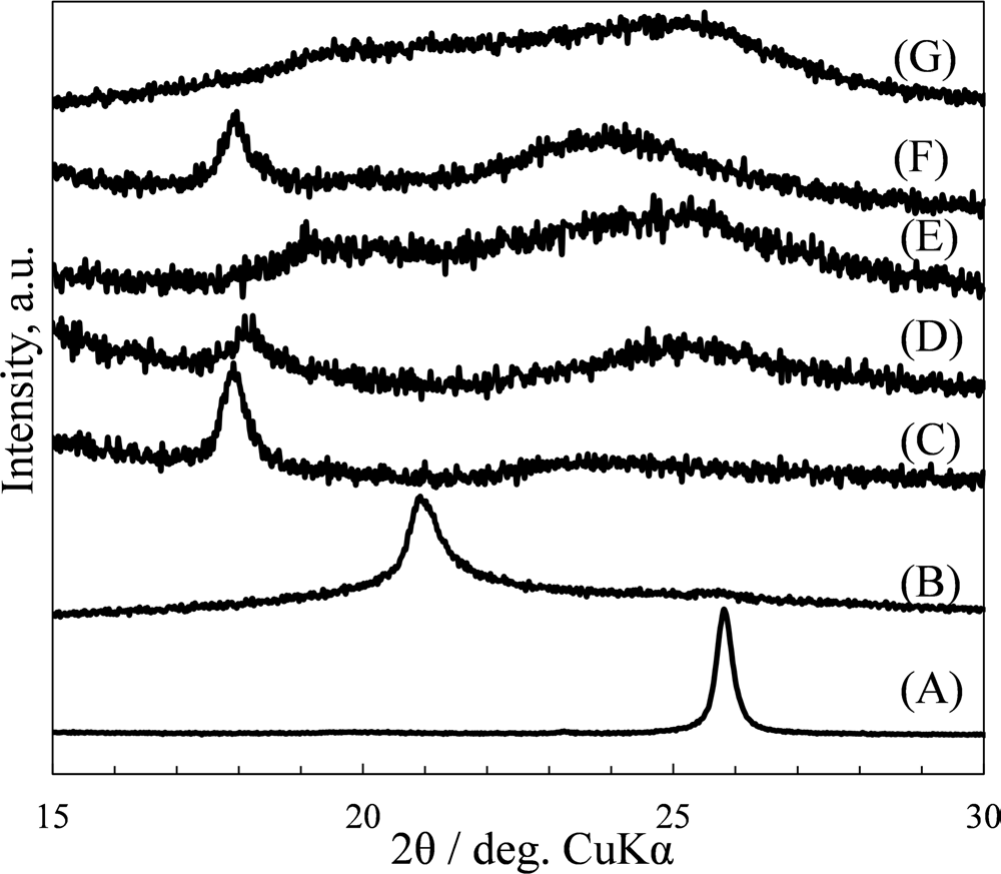
X-ray diffraction patterns of GLG. (**A**) Before and after charged or discharged to various cell voltages, (**B**) 0.48 V, (**C**) 0 V during 1^st^ charge (**D**) 1.2 V, (**E**) 2 V during 1^st^ discharge (**F**) 0 V during 2^nd^ charge, and (**G**) during 2^nd^ discharge.

*Ex-situ* X-ray diffraction patterns observed at various cell voltages are shown in Fig. 12. The diffraction peak at 2θ = 26.26° observed for pristine GLG (d = 0.339 nm) shifted to a lower angle of 21.52° (d = 0.413 nm) at 0.48 V then reached 18.44° (d = 0.481 nm) at 0 V, as observed in our previous studies^27,28,30^. This result clearly indicates that the Li ions are stored between the layers of GLG. During discharge, however, the diffraction peak shifted to a slightly higher angle of 18.75° (d = 0.473 nm) and became broader at 1.2 V, suggesting the de-intercalation of Li ions. The diffraction peak was still observed at a low angle of 2θ = 19.41° (d = 0.457 nm), even at 2 V, and became smaller, and a broad peak at 2θ = 25.3° (d = 0.352 nm), which was rather similar to that observed for pristine GLG, was observed. This suggests that some of the Li ions irreversibly remained in GLG. However, in the 2nd cycle, a sharp peak at 2θ = 18.48° (d = 0.480 nm) was again observed at 0 V, indicating that the intercalation of Li ions stayed in the same status compared with the 1st charge.

**Figure 13:**
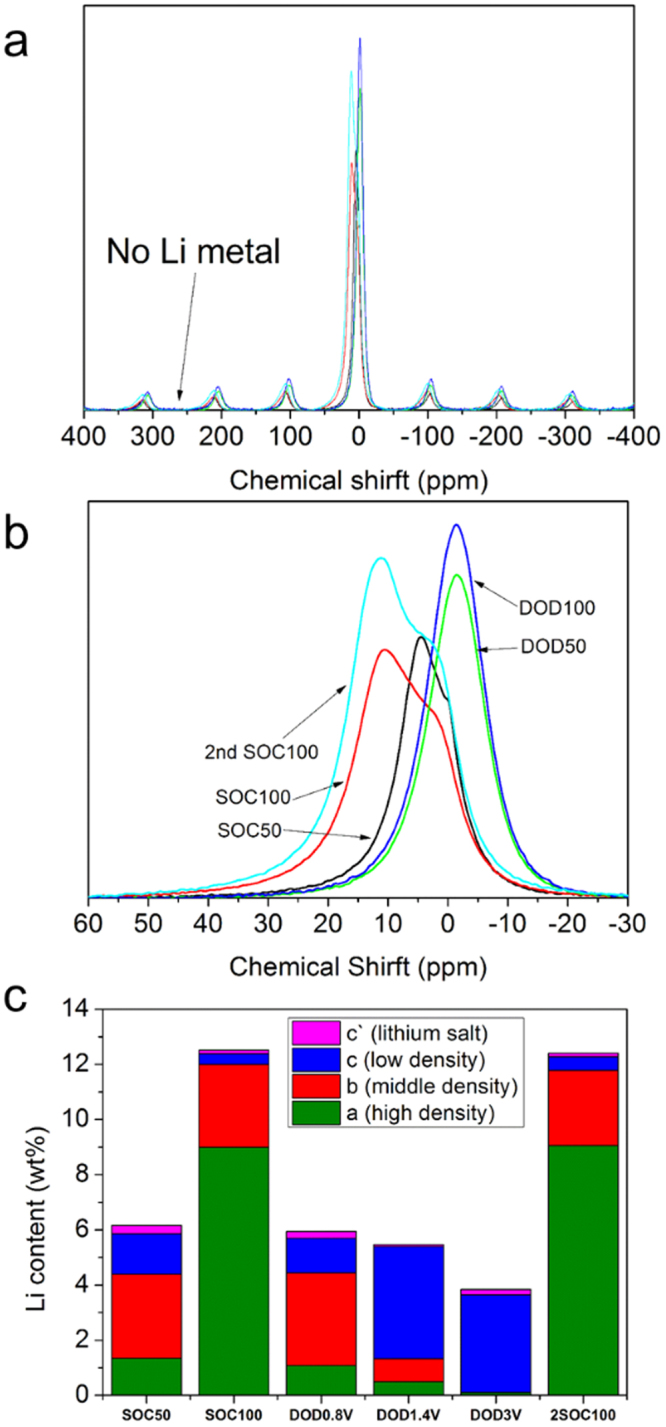
^7^Li NMR spectra of GLG at SOC50, SOC100, DOD50, DOD100, and 2^nd^ DOD100 states. (**b**) Enlarged spectra of (**a**). (**c**) Amount of Li ions in GLG of each state of charge determined from ICP measurement. Different colors mean contributions of each peak observed in ^7^Li-NMR spectra.

The ^7^Li-NMR spectra of GLG measured at SOC50, SOC100, DOD50, DOD100, and 2^nd^ DOD100 states are shown in Fig. 13. Even at the SOC100 state, no peaks around −260 and −46 ppm were observed, indicating that there was no deposition of Li metal or cluster, as observed for hard carbons^47,48^, and the state of Li ions in GLG differed from that in LiC_6_^49^. The peak position observed for GLG was rather similar to that observed for graphite with lower Li content or soft carbons prepared at lower temperatures^50^. In all cases, a broad peak was observed around 0 ppm and could be deconvoluted into 4 peaks at 11–12, 4–7, −1–1, and −1.4–1.5 ppm. Hereafter, these peaks are denoted as peaks “a”, “b”, “c”, and “c’”. To accommodate a large amount of such highly ionic Li ions, it is necessary to shield the coulombic force acting between them. Electronegative O atoms introduced in GLG could play this role. Figure 12c indicates the amounts of Li determined from inductively coupled plasma (ICP) measurement of the GLG electrodes. The bars are filled with four different colors, indicating the contribution of four ^7^Li-NMR peaks estimated from their areas. The contribution of peak “c’” was always small, and we assigned this peak to that from the Li ions in SEI because the thickness of the SEI layer is very small based on the XPS depth profiles of GLG at various states, as shown in Figs S2–S13. The Li filling density in GLG decreasing from peak “a” to peak “c” (a > b > c) depends on the chemical shrift^50^. The amount of Li ions was 6.1%, and the contribution of that providing peak “b” was the largest at the 1^st^ SOC50 state. Then, the amount of Li ions increased to 12.3%, and the contribution of peak “a” became dominant at the SOC100 state. At the DOD50 state (0.8 V), the amount of Li decreased to 5.9%, and the ratio of the four types of Li ions became almost identical to that observed at the SOC50 state. This means that most of the removed Li ions were those providing peak “a”. The amount of Li ions further decreased to 5.2% at 1.4 V. The contribution of peak “a” further decreased and that of peak “b” also decreased; instead, peak “c” increased. This suggests that a large amount of Li ions providing peak “b” are converted to those providing peak “c” as the result of the decrease in the Li content in GLG. At 3 V, the amount of Li ions decreased to 4%, and only peak “c” was basically observed. At 0 V in the 2^nd^ cycle, the same amount of Li ions and ratio of the contribution of each peak as those in the 1^st^ cycle were observed, as expected from the X-ray diffraction data. It is important to point out that no Li-metal growth, even charged to 0 V, can lead to good safety properties of GLG. As a result, it is expected that the capacity discharged below 0.8 V is related to peak “a”, while discharge capacity delivered above 0.8 V is related to peak “b”. Finally, peak “b” partially transforms to peak “c” as irreversible capacity during initial charge and discharge.

**Figure 14:**
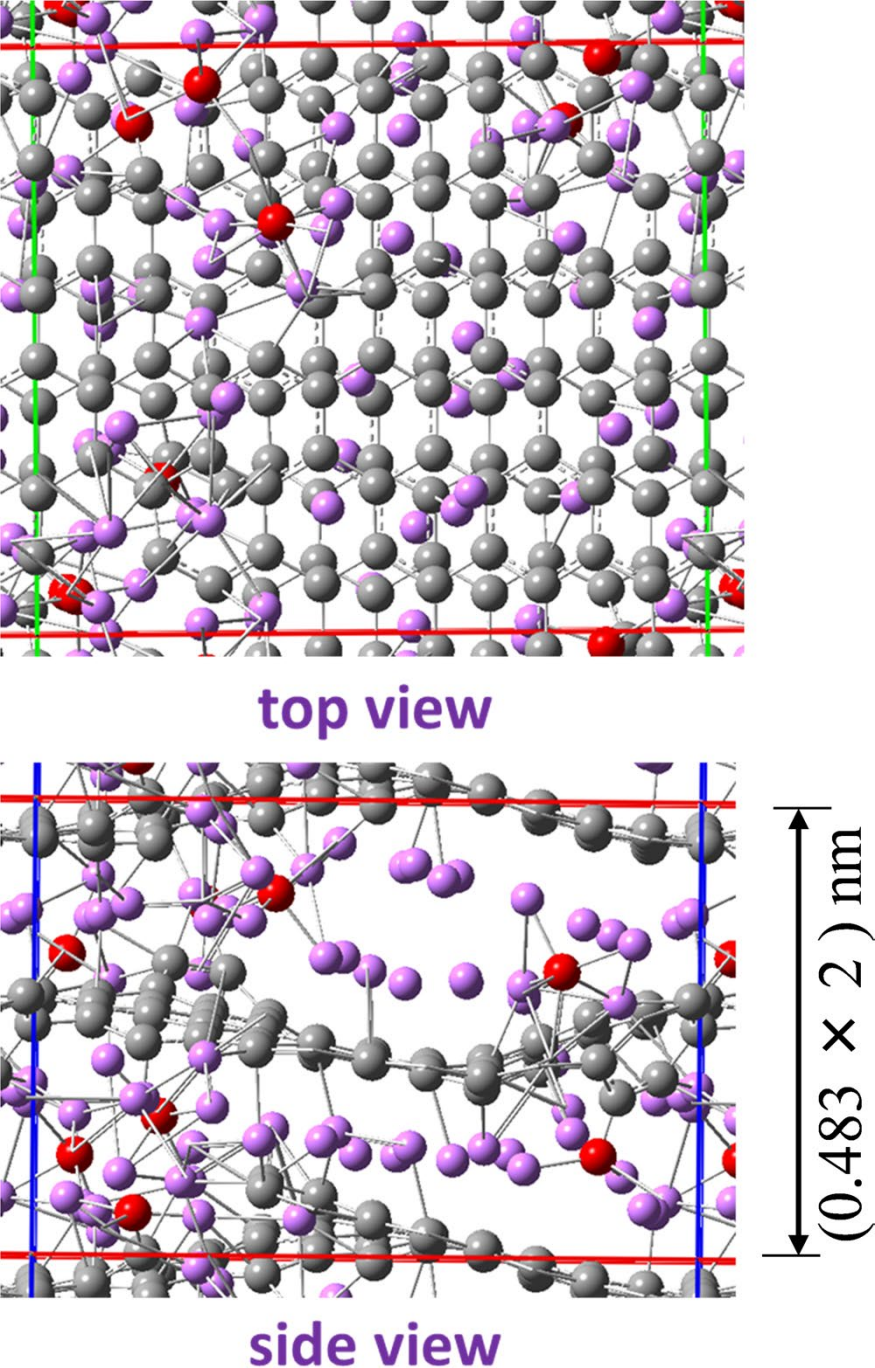
Optimized structure of Li_54_C_128_O_8_.

An atomic-level structure-model of lithiated GLG was examined using the first-principles calculations with periodic boundary conditions. The model contains 7.7 wt% of O atoms in substitutional sites of C atoms based on the model discussed in Section 3.1, and the O atoms at the edge of the nanopores were not considered. Figure 14 illustrates the model after the initial Li insertion. According to the total energy calculations of GLG with various Li content, lithiated GLG with the composition of Li_16_C_128_O_8_ or higher Li content was dischargeable below 1.2 V. This indicates that the theoretical charge capacity reaches 896 mAh/g or more. The larger increase in the interlayer spacing from 0.341 to 0.483 nm after lithiation was well reproduced, as observed for fully charged GLG (Fig. 12). Accordingly, this large increase in the interlayer spacing, resulting from the migration of O atoms within the carbon layer to the interlayer space though one of the C-O bonds, was still maintained. Lithium ions were found in the vicinity of both O and C atoms, and the number of Li ions, which interact with O atoms, was much larger. This suggests that the high O content in GLG is responsible for its large capacity exceeding the theoretical capacity of graphite, 372 mAh/g. Considering that the larger electronegativity of O than C, the peaks observed around 0 ppm in ^7^Li NMR (peaks “c”) may be due to Li ions interacting with O atoms as C-O-Li_(n)_. Peak “b” is attributed to C-Li at around 6 ppm, and peak “a” around 10 ppm is therefore attributed to the Li ions located between the carbonaceous interlayer as C-Li_(n)_. This idea seems reasonable because, among the three types of Li ions introduced in soft carbons, that removed at 0.12–0.8 V provides the peak at a similar position.

**Figure 15:**
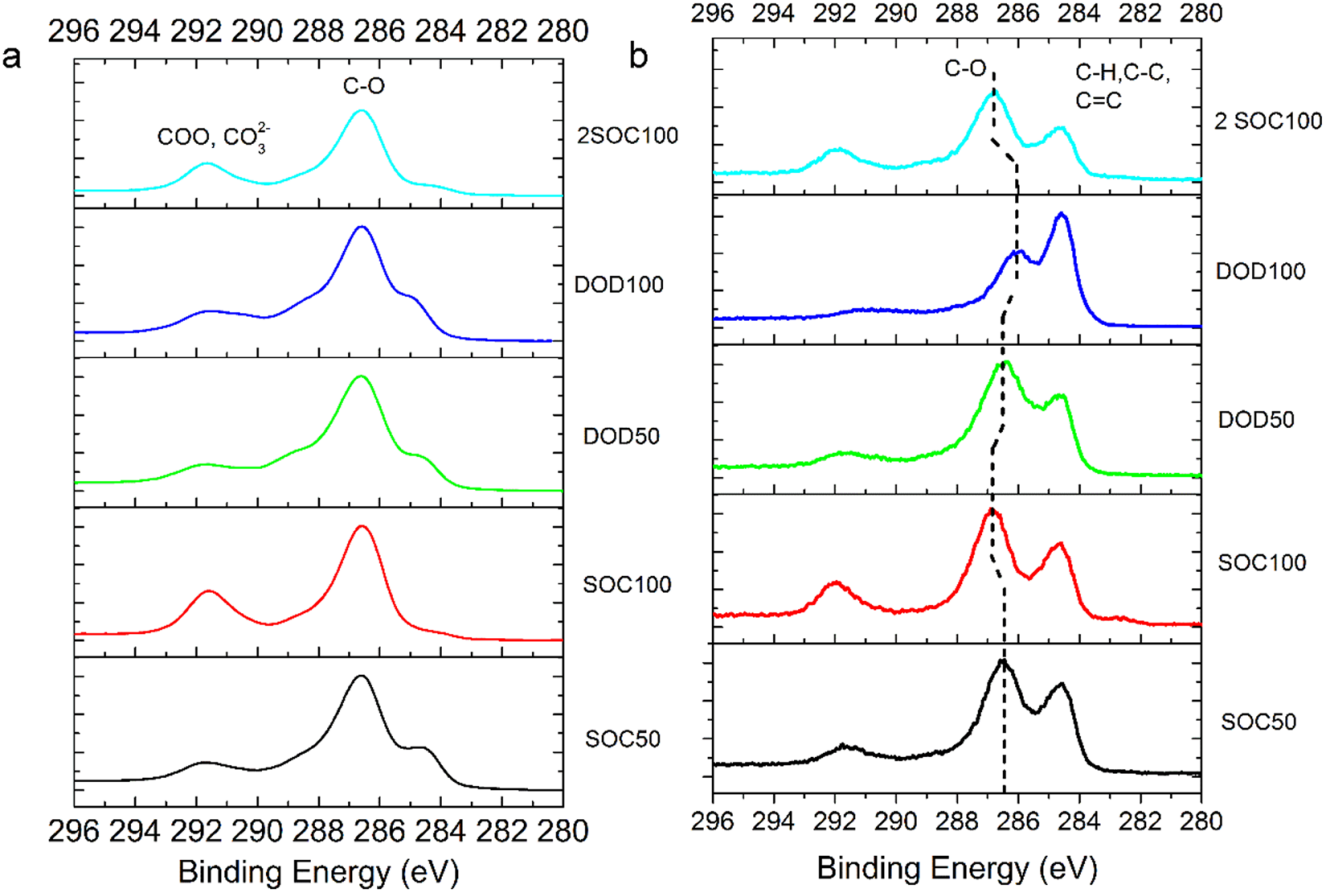
XPS (**a**) and HAXPES (**b**) results of GLG electrode at SOC50, SOC100, DOD50, DOD100, and 2^nd^ SOC100 states.

Figure 15 shows the XPS and HAXPES spectra of a GLG electrode at various charge or discharge states. In XPS, peaks at 292, 286, and 284 eV were observed, which are assigned to COO^−^ and CO_3_^2−^, C-O and C-C, and C-H and C=C bonds, respectively^51^. The former two peaks should be attributed to the SEI, and their relative intensity increased during charge and decreased during discharge^52^. This means that the thickness of SEI changes during charge and discharge processes, which is different from the case with a graphite-based anode^52–57^. As shown in the depth profiles of C, Li, O, F, and P (Figs S2–S13), the peaks due to SEI became smaller as the sputtering time increased, and its thickness was estimated to be 32 nm for GLG at the 1^st^ SOC100 and 2^nd^ SOC100 states, and 18 nm for that at the DOD100 state. In HAXPES, as shown in Fig. 15b, the above peaks at 292, 286, and 284 eV were also observed, and its relative intensity at 284 eV due to C = C in GLG became larger, reflecting the larger invasion depth of hard X-ray (~50 nm) and thin SEI. The peak at 286.4 eV shifted to a higher binding energy to reach 287 eV at the SOC100 state then shifted to a lower binding energy to reach 286.0 eV at the DOD100 state. The shift of this peak to a higher binding energy was also observed during the 2nd cycle. The shift of the peak around 286.5 eV was not observed when the GLG electrode was analyzed by XPS, suggesting the change in environment of C-O bond in GLG as a result of the binding it with Li ion(s).

## Conclusion

We designed and fabricated a C-based anode material called GLC with similar morphology and interlayer spacing to those of graphite. It exhibited a high capacity of 608 mAh/g with an upper limit cell voltage of 2 V as well as much higher capacity at 6 and 10 C than pristine graphite. Based on various analytical techniques such as XRD, XPS, HAXPES, RBS, TEM, and SEM observations, we found that O atoms are mainly introduced in the state of C-O-C within the carbon layers, which are regularly stacked to form graphite-like particles. The Li ions are stored between the GLG layers and, according to the ^7^Li NMR data obtained for GLG at various charge or discharge levels, three types of highly ionic Li ions were found. A large increase in the interlayer space of 0.14 nm was observed for fully charged GLG. Theoretical calculation indicated that the migration of O atoms to the interlayer space maintaining one of the C-O bonds is responsible for this increase. Some of the introduced Li ions remained in GLG even after discharged to 3 V, forming a stable structure, which was confirmed by the almost identical X-ray diffraction patterns observed for fully charged and discharged GLG in the 1st and 2nd cycles. This explains the high stability during charge-discharge cycling. These results strongly indicate that GLG is a promising C-based material for next-generation Li-ion battery anodes with both high capacity and fast-chargeable capability for electric vehicles and plug-in hybrid vehicles with autopilot capability. For the next step, we will attempt to optimize the O content, surface coating, hetero-atom doping, etc, to improve capacity, coulombic efficiency, and rate performance.

## Electronic supplementary material

Supplementary info
