# Supplementary material for: Graphene-Like-Graphite as Fast-Chargeable and High-Capacity Anode Materials for Lithium Ion Batteries
Source: Sci Rep. 2017 Nov 1;7:14782. doi: 10.1038/s41598-017-14504-8 (PMC5665891; doi:10.1038/s41598-017-14504-8)
Supplement: Supplementary file 1 — Supplementary info [file 41598_2017_14504_MOESM1_ESM.doc]

Supporting Information

Graphene-Like-Graphite as Fast-Chargeable and High-Capacity Anode Materials for Lithium Ion Batteries

Qian Cheng^a^*, Yasuharu Okamoto^a^, Noriyuki Tamura^a^, Masayoshi Tsuji^a^, Shunya Maruyama^b^, Yoshiaki Matsuo^b^*

<mailto:>qchenghit@gmail.com

ymatsuo@eng.u-hyogo.ac.jp

a. IoT Devices Laboratories, NEC Corporation, Tsukuba, Ibaraki, 305-8501, Japan

b. Department of Applied Chemistry, Graduate School of Engineering, University of Hyogo, Himeji, Hyogo, 671-2280, Japan


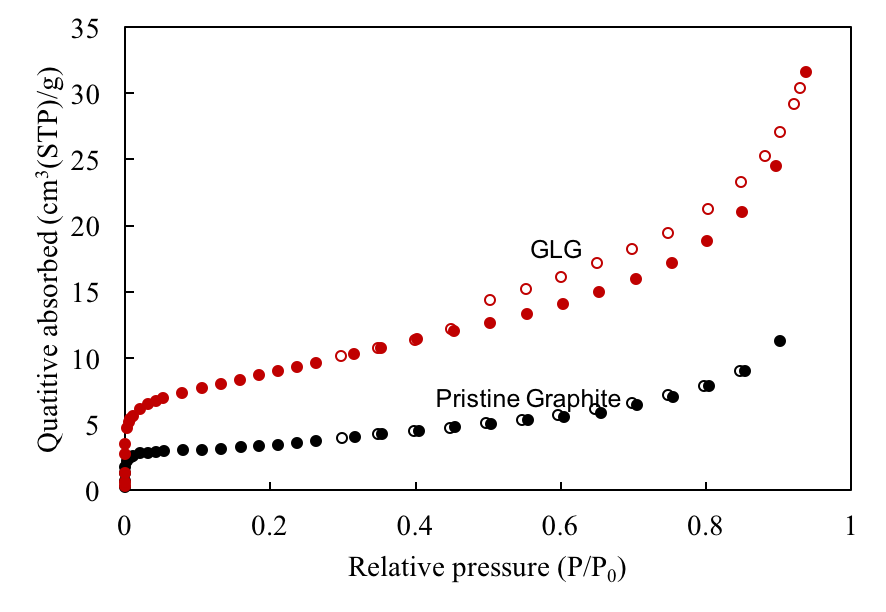


Figure. S1 Isotherm curves of pristine graphite and GLG.

Figure. S2 C1s XPS depth profile of SOC100

Figure. S3 C1s XPS depth profile of DOD100

Figure. S4 C1s XPS depth profile of 2^nd^ SOC100

Figure. S5 O1s XPS depth profile of SOC100

Figure. S6 O1s XPS depth profile of DOD100

Figure. S7 O1s XPS depth profile of 2^nd^ SOC100

Figure. S8 F1s XPS depth profile of SOC100

Figure. S9 F1s XPS depth profile of DOD100

Figure. S10 F1s XPS depth profile of 2^nd^ SOC100

Figure. S11 L1s XPS depth profile of SOC100

Figure. S12 L1s XPS depth profile of DOD100

Figure. S13 Li1s XPS depth profile of 2^nd^ SOC100


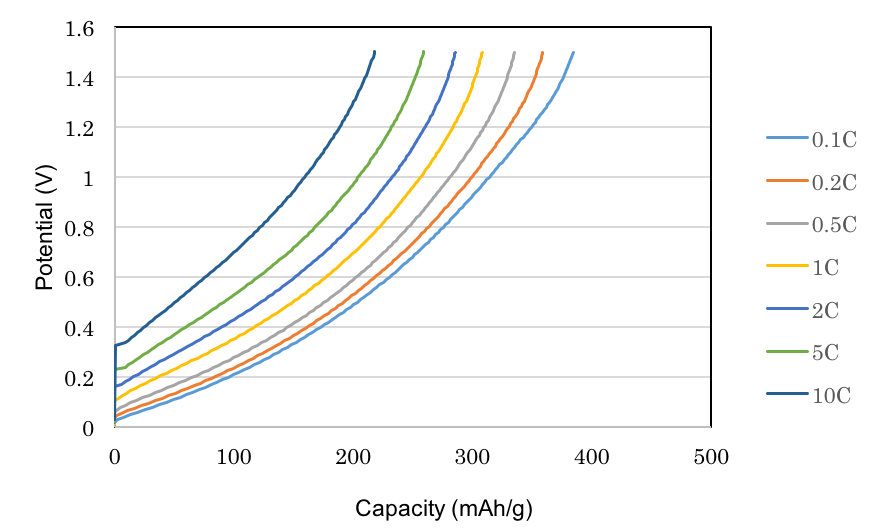


Figure. S14 Half-cell discharge curves of GLG in different rate


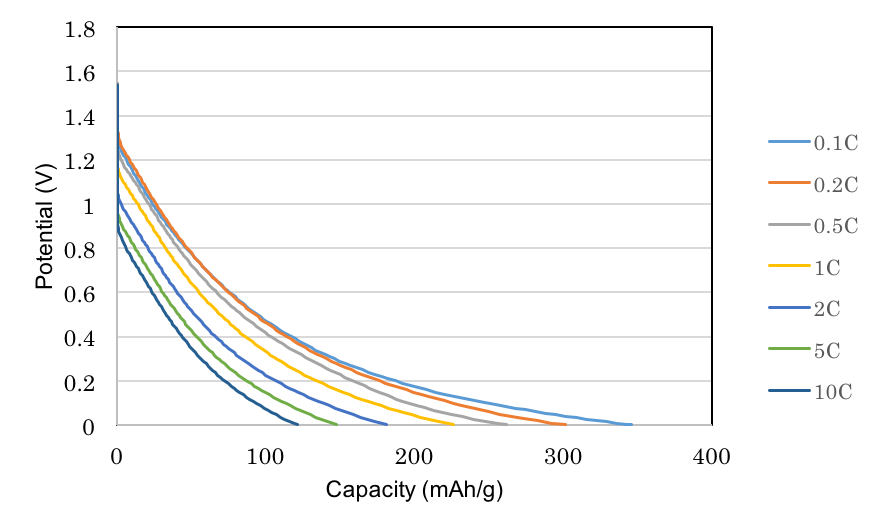


Figure . S15 Half-cell charge curves of GLG in different rate


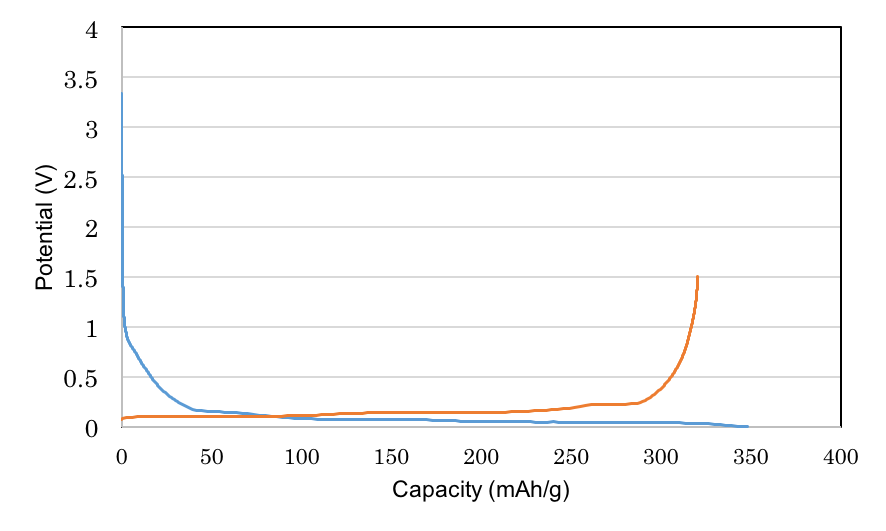


Figure S16 The charge and discharge curve of conventional graphite The coulombic efficiency is 92%


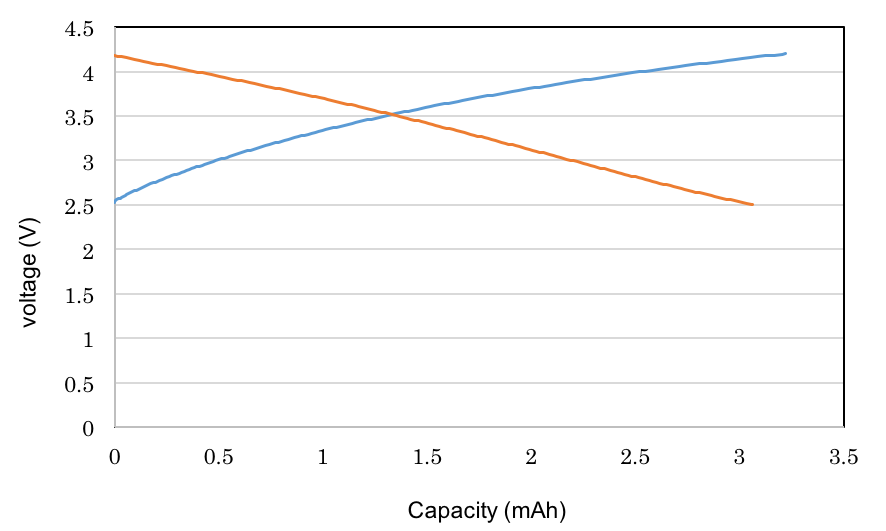


Figure S17 The charge and discharge curve of full cell GLG with NCM111


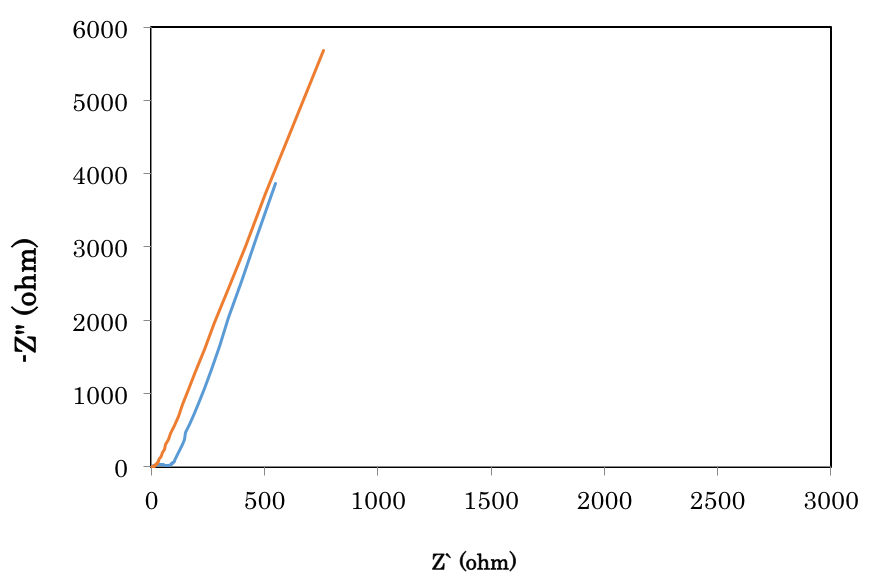

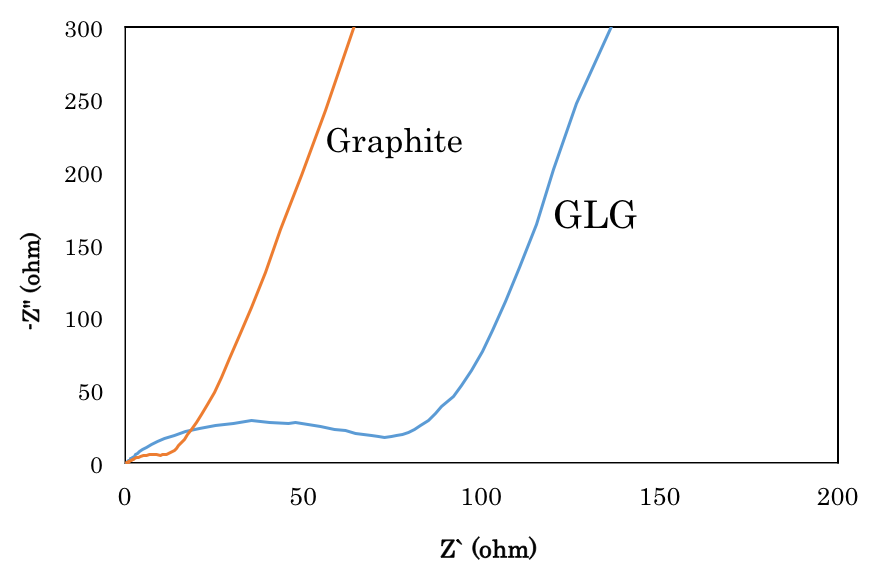


Figure S18 EIS curves of graphite and GLG
